# Application of a novel shape-memory alloy concentrator in displaced olecranon fractures: a report of the technique and mid-term clinical results

**DOI:** 10.1186/s13018-020-01982-2

**Published:** 2020-10-02

**Authors:** Demeng Xia, Panyu Zhou, Lei Li, Yan Xia, Zichen Hao, Yuntong Zhang, Shuogui Xu

**Affiliations:** 1 Department of Emergency, Changhai Hospital, the Naval Medical University, No.168, Changhai St, Shanghai, 200433 People’s Republic of China; 2 Department of Orthopaedics, Eastern Theater Naval Hospital, No. 98, Wenhua St, Zhoushan, Zhejiang, 316000 People’s Republic of China

**Keywords:** Olecranon fractures, Shape-memory alloy concentrator, Technique

## Abstract

**Purpose:**

Olecranon fracture is a common upper limb fracture, and several surgical approaches have been advocated for its fixation. To overcome the complications associated with common techniques, we present a novel shape-memory alloy concentrator, an alternative for tension band compression, to fix olecranon fracture.

**Methods:**

Fifty-seven patients (26 men and 31 women) with olecranon fracture, with a mean age of 45 years, were included in this study. Each patient had undergone open reduction and internal fixation using the Nitinol (Ni-Ti) arched shape-memory connector (ASC). The clinical assessments were performed using the Disability of the Arm, Shoulder, and Hand (DASH) questionnaire and the Mayo Elbow Performance (MEP) score, which were both recorded at the final follow-up visit.

**Results:**

The patients were followed up for 44 months on average (range, 31 to 56 months). No patients were lost to follow-up, and all of the olecranon fractures healed in an average of 15 weeks (range, 10 to 34 weeks). The mean DASH score was 8.6 (range, 0 to 32.4), and the mean MEP score was 92.5 (range, 74 to 100). Nine patients showed postoperative complications: prominent hardware (2), infection (1), loss of the range of functional motion (5), and heterotopic ossification (1).

**Conclusion:**

The ASC may serve as a favorable device for multi-fragmented and comminuted fractures with rare hardware irritation and may also provide continuous concentrative compression to accelerate osseous healing, thereby aiding the restoration and permitting an early rehabilitation with a low incidence of postoperative complications.

## Introduction

Olecranon fractures are among the most common injuries, accounting for approximately 1% of all skeletal injuries and up to 10% of fractures of the elbow joint [1–3]. Based on a literature review, only 5–7% of patients who had non-displaced or displaced fractures required manipulative reduction and external fixation; however, more than 93% of fractures that included fragment dislocation required open reduction and internal fixations [4, 5]. It has been reported that displaced transverse fractures of the olecranon are the most common fractures occurring in the elbow in adults that require operative intervention [6]. Although tension band wiring (TBW) is a well-considered standard to treat olecranon fractures [7], the approach, due to back-out or proximal migration of the longitudinal wires, stimulates soft tissues and may be a source of discomfort for the patients [8, 9]. On average, the rates of removal following TBW are reported to be as high as 80% [1]. Additionally, the tension band wiring method does not apply to all types of fractures [10]. Plate fixation (PF) can overcome the main shortcoming of TBW and is mainly reserved for comminuted fractures or distal fractures, showing a biomechanical advantage over tension-band wiring and a reported lower rate of hardware removal [11]. Nevertheless, extreme bending stresses at the proximal part of the ulna can lead to fatigue failure of internal fixation devices occasionally [12]. Therefore, a novel surgical method to treat olecranon fractures has always been explored.

First appearing in the 1960s, nitinol (Ni-Ti) shape-memory alloys were approved by the FDA for clinical application in 1990. These alloys seem to open a whole new range of applications due to their shape-memory effect, corrosion resistance, wear resistance, super-elasticity, and favorable histocompatibility [13–16]. This innovative material has been widely used in the neurosurgery [13], cardiovascular [17], and orthopedic fields [18]. To further utilize the special mechanical behavior of the shape-memory material, we designed a shape-memory alloy concentrator called the Ni-Ti arched shape-memory connector (ASC), which has been applied for internal fixation since the 1990s [19–21]. The current study aimed to introduce the peculiarity of the ASC and retrospectively evaluate the efficacy and clinical outcomes of the ASC in the treatment of olecranon fractures.

## Materials and methods

**Fig. 1:**
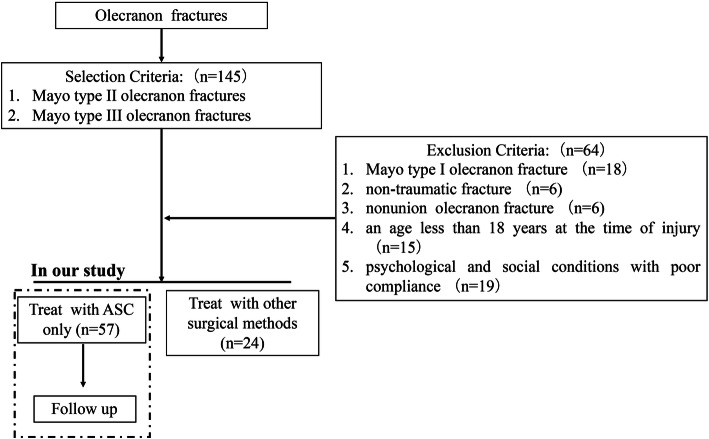
The flowchart representing selection and exclusion for analysis of ASC for olecranon fractures

One-hundred and forty-five patients with olecranon fractures, who were admitted to the Trauma and Emergency Department of Changhai Hospital, Naval Medical University, between December 2010 and December 2017, were recruited for this study. The Committee on Ethics of Biomedicine Research of Changhai Hospital authorized all the procedures. The selection criteria included patients with Mayo type II and type III olecranon fractures. The exclusion criteria included patients with (1) Mayo type I olecranon fracture, (2) non-traumatic fracture, (3) nonunion olecranon fracture, (4) an age less than 18 years at the time of injury, and (5) psychological and social conditions with poor compliance. The flowchart representing the selection and exclusion for the analysis of ASC for olecranon fractures is shown in Fig. 1.

### Structure and working principles of the ASC

The ASC device (Huzhou Swan Biological Memory Medical Devices Co., Ltd., Zhejiang, China) is designed based on the anatomical structure of the olecranon and manufactured with 2-mm-thick Ti-Ni shape-memory alloy. The ASC comprises of 50–53% nickel, with the remainder comprising titanium. The device has compression arms that are connected to the waist. The transition temperature is set at 33 °C ± 2 °C with one-way heat treatment. The device exhibits a unilateral memory effect and a shape change quantity of 8%. The ASC is malleable at lower temperatures (martensite phase); thus, it is placed in 0–4 °C ice water for cooling before implantation. When the fixation is completed, 40–50 °C water was used to warm the device to stimulate its mechanical memory functions (austenite phase), providing a continuous lateral compressive force. The shape change process of the ASC is shown in video 1 as supporting materials

### Surgical procedure

**Fig. 2:**
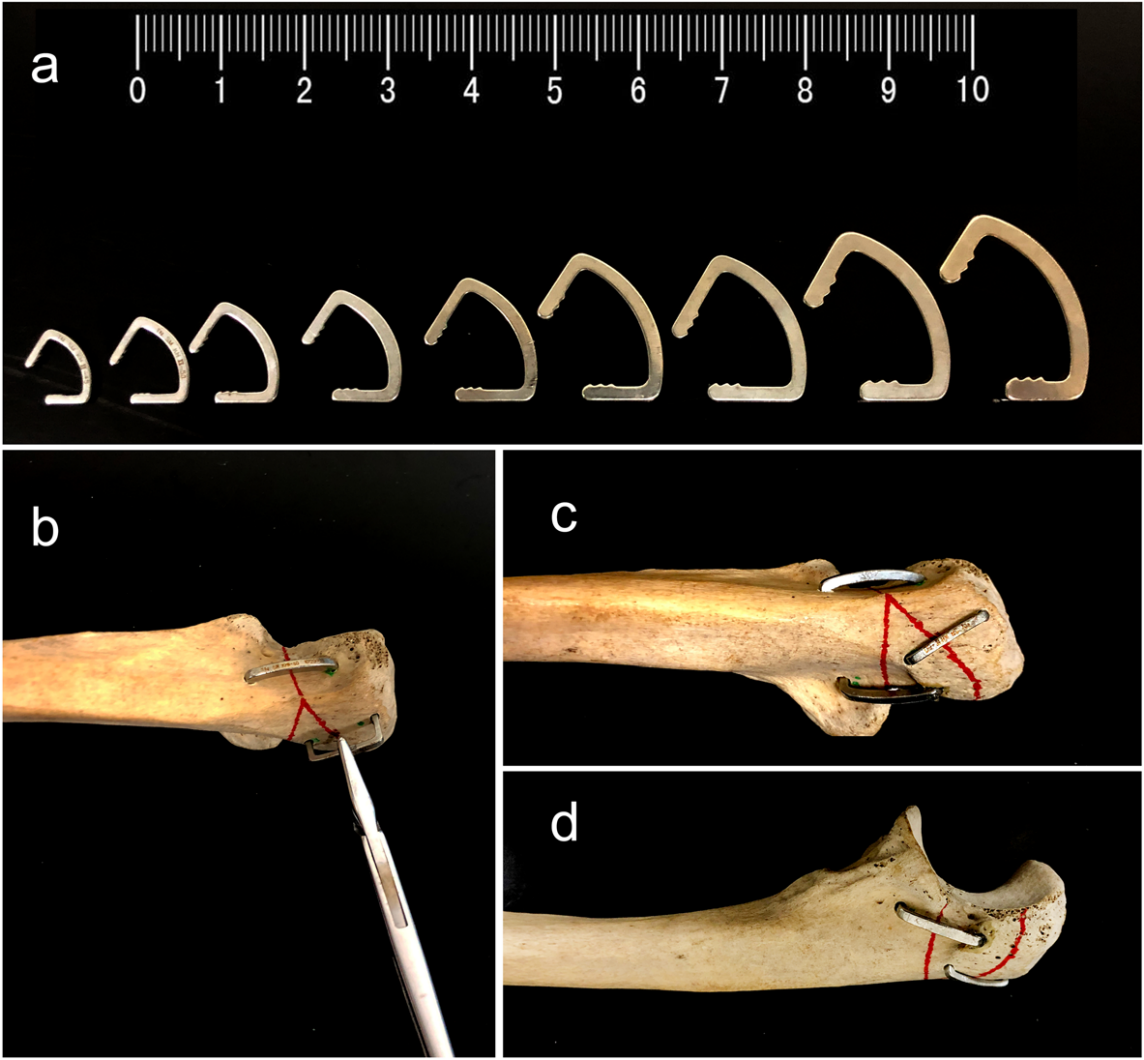
Different size of arched shape-memory connectors which can be applied on different types of fractures, choose the most suitable arched shape-memory connector (**a**). Drill two holes on both sides of the fracture line and place the arched shape-memory connector vertically according to the fracture line (**b**). Finally, embed the compression arms and reheated in warm water to stimulate the reversion of the arms and waist back to their original shapes; several arched shape-memory connectors are used for the stable fixation of fractures and diagrams of the arched shape-memory connector fixation on specimen in AP (**c**) and lateral (**d**) views

A longitudinal posterior skin incision was made to the exposure of the olecranon. Excessive exposure of the dorsal surface of the proximal ulna was not recommended only when the comminuted fracture lines were involved. If any articular fragment was impacted, it was elevated, and any coronoid fracture was reduced and temporarily fixed to the ulna with Kirschner wires. After clearance of the bone debris and soft tissues and internal fixation of the olecranon fracture, the ASC was preincubated previously in ice water (0–4 °C) to allow the plastic deformation of the Ni-Ti alloy. Next, the arm was unfolded using needle forceps, and two holes were drilled on either side of the fracture lines to embed the arms of the ASC, where the ligature between the two holes was as perpendicular to the fracture line as possible. According to the actual condition of the ulnar fracture, two or more additional ASCs were required for stable fixation. After the fracture sites were reduced, the ASC was embedded into the bone and reheated in warm water (40–50 °C) to stimulate the reversion of the arms and waist back to their original shapes, which correspondingly generated a continuous compression force on the fracture line. Based on the above procedures, the memory alloy created fixation by maintaining the bone block in a system driven by temperature. The application process of the ASC is briefly illustrated in Fig. 2, and detailed introduction of the structure and working principles can be reviewed in previous reports [22, 23]. The subcutaneous tissues and skin were closed in the usual way of surgery. Finally, a removable plaster stand was used to hold the elbows bent to 90°. If pain could be tolerated, the patient was encouraged to make active movements of the fingers and isometric contraction of the upper arm muscles. From the second day after the operation, active assistive exercise was gradually performed. At the same time, the patient was required to remove the arm from the plaster support, and the flexion and extension of the elbow were performed appropriately several times a day, gradually increasing the amplitude and range of the movement.

### Clinical outcomes evaluation

The follow-ups were performed, and radiographic assessments were routinely performed at 1 month, 2 months, 3 months, 6 months, and 1 year and at a half-year or 1-year interval thereafter. The Disability of the Arm, Shoulder and Hand (DASH) questionnaire was used to evaluate impairments and activity limitations [24]. The Mayo Elbow Performance (MEP) score was also used as a comprehensive assessment considering the following four factors: pain, ulnohumeral motion, stability, and the ability to perform five functional tasks [24]. According to the previous report [25], the functional range of movement in the operated elbow was flexion ≥ 128°, extension ≥ 116°, and pronation and supination ≥ 72°; when any one of the elbow joints does not meet these 4 criteria, we considered it a loss of the functional range of motion. All the clinical assessments used in this study were performed by independent observers, and the results were independently interpreted by two authors.

## Results

Of the total number of patients recruited for the study, 57 patients (26 men and 31 women) were eligible for inclusion in the research. The mean age of the patients was 45 years (range, 22–60 years). The main cause of fracture in 23 (40.4%) of the patients was a fall. Meanwhile, the mechanism of injury in 19 (30.3%) of the patients was a vehicular accident, whereas that in the remaining 15 (26.3%) patients was a sports-related injury. The classification of all of the fractures from the patients was made based on the basis of the Mayo classification system, which distinguishes the following three factors that directly influence on treatment: fracture displacement, presence of comminution, and ulnohumeral stability [2]. In this study, 14 fractures (24.6%) were type IIA, 14 fractures (24.6%) were type IIB, 20 fractures (35.1%) were type IIIA, and 9 fractures (15%) were type IIIB. The demographics of olecranon fracture patients are shown in Table 1.

**Table 1:** Demographics of olecranon fracture patients

Patients, *n*	57
Age, years(range)	45 (22–60)
Sex, *n* (%)	
Male	26
Female	31
Mechanism of injury, *n* (%)	
Fall onto elbow	23
Vehicular accidents	19
Sports injury	15
Mayo classification	
Type IIA	14
Type IIB	14
Type IIIA	20
Type IIIB	9
Mean time to surgery, days (SD; range)	1.9 (3.2; 1–7)
Mean operative time, mins (SD; range)	68 (11.2; 35–133)

None of the fractures were open injuries. Thirty patients sustained other fractures at the time of olecranon injury, including three radial head fractures, three coronoid fractures, three calcaneal fractures, two pelvic fractures, two femoral fractures, and one tibial fracture. The mean duration of the operation was 1.9 days (range, 1 to 7 days) from the time of the initial injury (Table 1). The mean duration of the operative time spent dealing with the olecranon fracture was 68 min (range, 35 to 133 min).

**Fig. 3:**
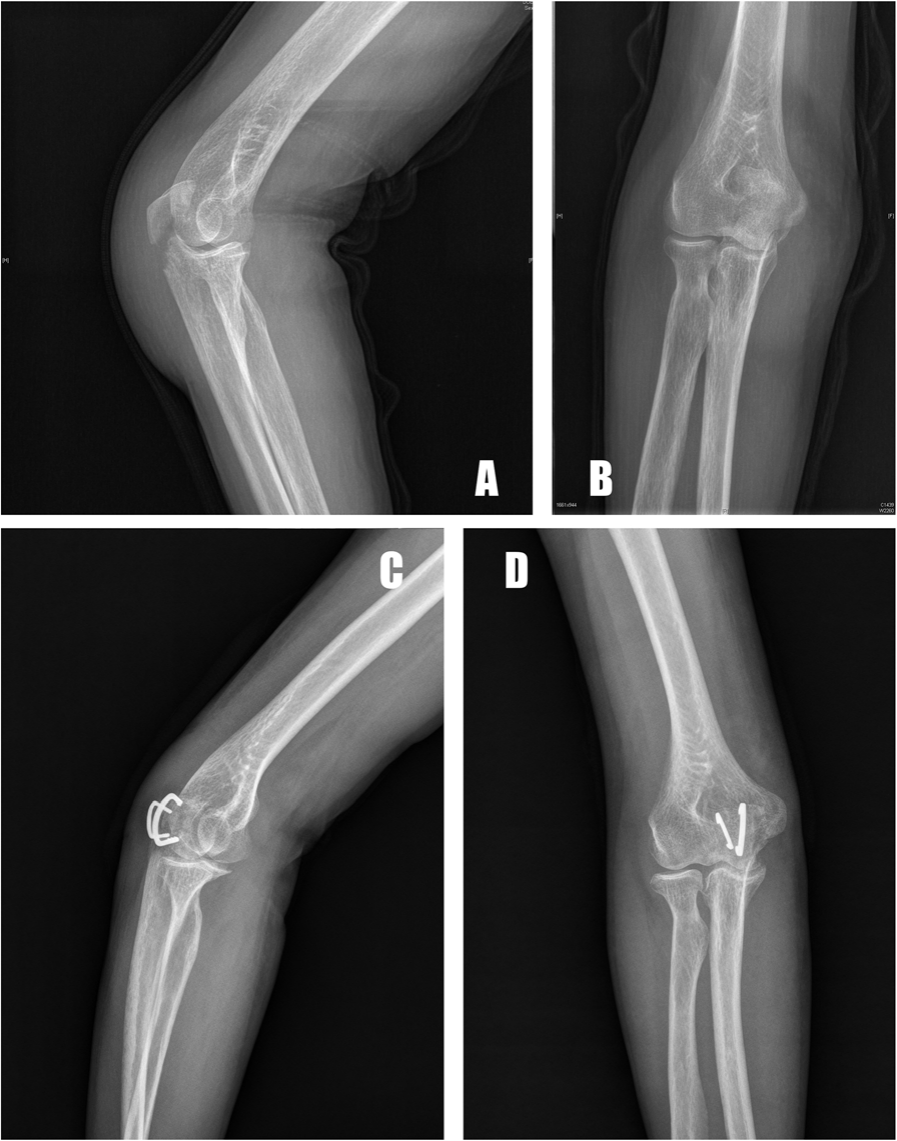
A 54-year-old male patient with comminuted olecranon fracture caused by a vehicular accident (**a**, **b**). The comminuted fragments were reduced and fixed anatomically with two ACS. Seven months after surgery, the radiographs showed a healed fracture with metallic implants and no obvious gap or step-off along the joint surface (**c**, **d**)

**Fig. 4:**
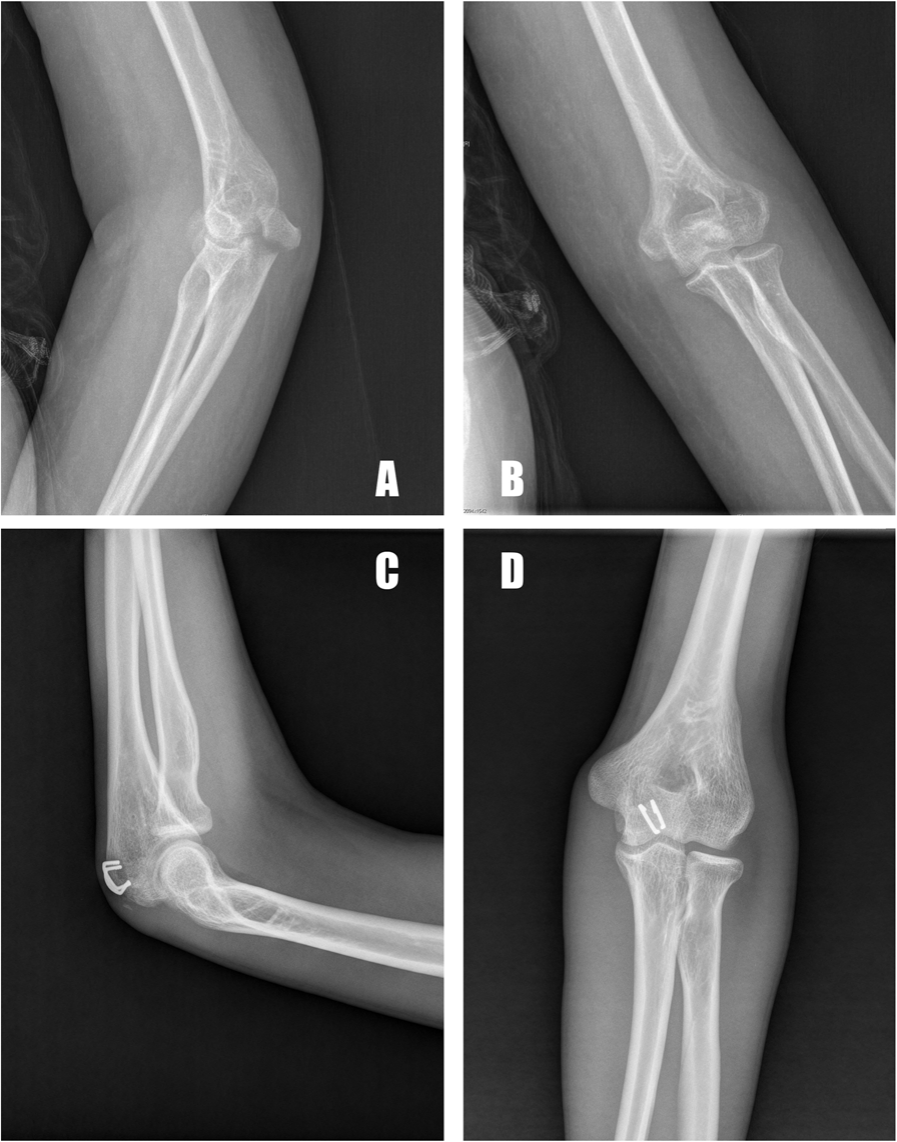
A 38-year-old male patient with olecranon fracture caused by a fall onto his ankle (**a**, **b**). Fragments were reduced and fixed with the ASC. Ten months after the surgery, the radiographs showed a healed fracture and the ankle joint surface was anatomically reduced without any gap or step-off (**c**, **d**)

**Fig. 5:**
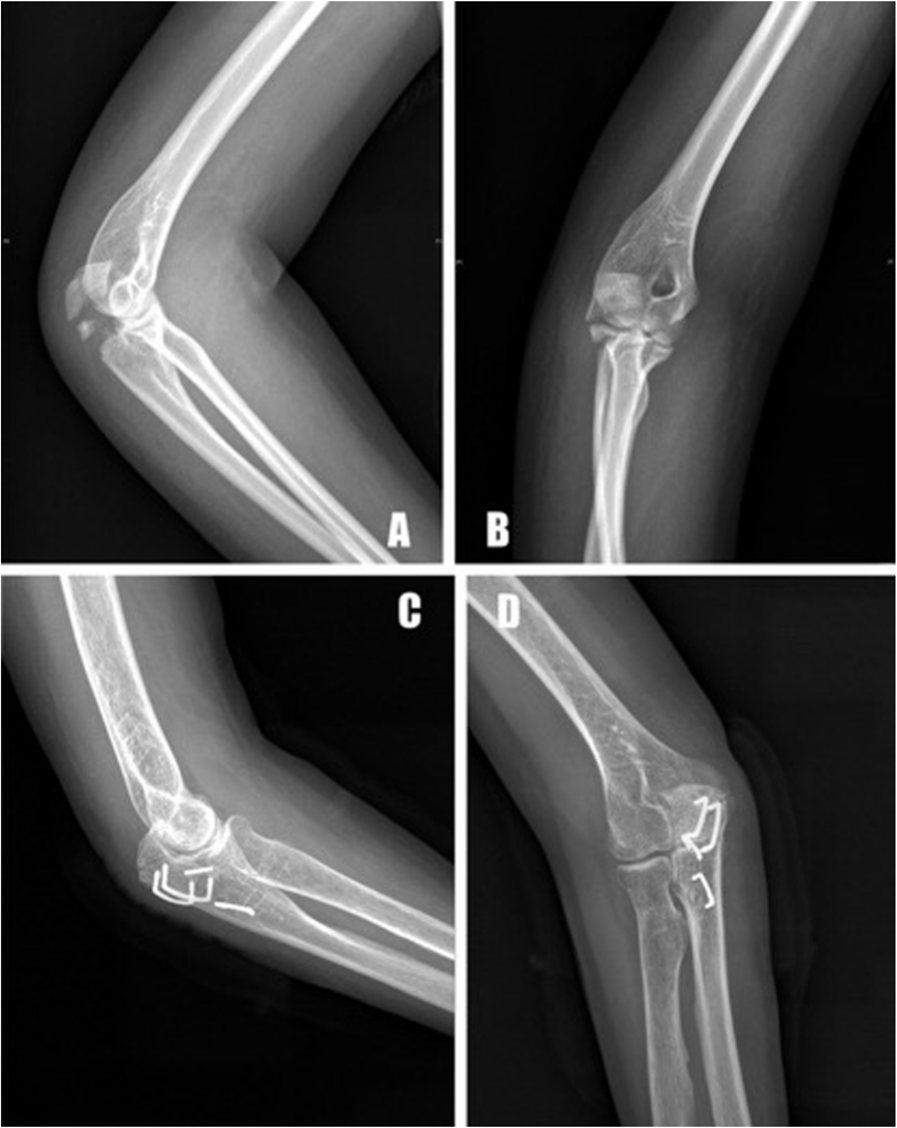
A 55-year-old male patient with commuted olecranon fracture caused by a traffic accident (**a**, **b**). Fragments were reduced and fixed with the four ASCs. Eleven months after the surgery, the radiographs showed healed fractures, and the ankle joint surface was anatomically reduced without any gap or step-off (**c**, **d**)

The postoperative radiographic measurements demonstrated an anatomical or nearly anatomical reduction of olecranon fractures in all patients (Figs. 3, 4, 5). None of the patients showed a postoperative articular gap of more than 2 mm. The patients were followed up for 44 months on average (range, 31 to 56 months). No loss of anatomical reduction was observed in the patients, and all of the olecranon fractures healed after an average of 15 weeks (range, 10 to 34 weeks). At the most recent follow-up, the mean DASH score was 8.6 (range, 0 to 32.4), and the mean MEP score was 92.5 (range, 74 to 100). Nine patients had postoperative complications: prominent hardware (2), infection (1), loss of range of functional motion (5), and heterotopic ossification (1), and it was obvious that the most common complication was the loss of functional motion. Mild pain due to prominent hardware was reported in one patient who had two ASCs implanted at the dorsal of the olecranon due to the presence of the proximal pole fracture line, but it was well-tolerated. Furthermore, none of the patients requested metal removal during the follow-up period. The preoperative and final follow-up clinical outcomes are presented in Table 2.

**Table 2:** Preoperative and final follow-up clinical outcomes

Follow-up time, months (range)	44 (31–56)
Postoperative complications, *n*	
Prominent hardware	2
Infection	1
Arthritis	0
Loss of range of functional motion	5
Flexion < 128°	3
Extension < 116°	1
Pronation < 72°	1
Supination < 72°	0
Heterotopic ossification	1
Nonunion	0
Nard failure	0
DASH scores (SD)	8.6 (2.1)
MEP scores (SD)	92.5 (7.5)

## Discussion

Fifty-seven patients were followed up, and all of the olecranon fractures healed in an average of 15 weeks in our study. The mean DASH score and the MEP scores were favorable. However, 9 patients had postoperative complications, the most common being the loss of functional motion.

The aims of treatment are to provide absolute stability of the fracture and to restore the joint surface to allow range-of-motion exercises in the early postoperative period [26]. Compared with the rare nondisplaced fractures with a displacement of less than 2 mm, for which non-operative treatment is recommended [27], open reduction and internal fixation are recommended for most olecranon fractures to avoid complications such as fracture nonunion, joint stiffness, traumatic arthritis, ossifying muscle inflammation, and joint adhesions [28]. In clinical practice, various internal fixations can be applied to reconstruct the anatomical structure and recover the elbow function, such as figure-eight steel wire fixation, anatomical plate, K-wire tension band, hollow nail plus tension, and 1/3 tube type plate [19, 29, 30], the most frequently used techniques being tension band wiring (TBW) and plate fixation (PF) [28, 31].

The technical advantage of TBW is that this method can convert tension at the fracture site, which is produced by tension band fixation into compression forces at the articular surface, making the fracture site closer and accelerating the healing of the fracture [32]. Furthermore, TBW allows for less soft-tissue stripping, which contributes to less damage [33]. Compared with TBW, PF possesses the advantage of an increased stability [31] and is related to lower rates of hardware prominence [34]. Luksic et al. [25] reported that in the treatment of comminuted olecranon fractures a new technical tension plate combined with two Kirschner wires could provide adequate stability for early postoperative functional recovery, producing good clinical outcomes and increasing the fracture union rate [25]. However, hardware-related symptoms have been a common complication, with a rate of approximately 56 to 76.2%, leading to a secondary operation for removal [1, 12]. Additionally, the excessive strip of periosteum and intraoperative damage of soft tissues will affect the healing of fractures.

Despite the lack of long-term rigorous controlled studies, our surgical team usually prefers the application of the ASCs for Mayo type II and type III olecranon fractures without nonunion or non-traumatic fractures when the patients tolerate the operation. The ASC used in this study is an industrial product into which the Ni-Ti shape-memory alloy is incorporated. The ASC has the biocompatibility and corrosion resistance superiority of the Nitinol shape memory alloy and can transform its shape with temperature changes, improving the intraoperative and postoperative outcomes. Although the ASC has been effectively applied to treat the humeral shaft, acetabular, and scaphoid waist fractures [19, 20, 35–37], no clinical study has been reported on its use in the treatment of olecranon fractures. Applying an optimal technique may not only reduce the postoperative complications but also decrease the need for secondary operations, particularly metal removal, and patients’ discomfort. Compared with conventional techniques, the ASC may have several advantages. (1) Due to the limited space and thin skin at the olecranon, traditional implants will cause skin irritation and pain. Regardless of whether TBW and PF or other methods are used, the hardware removal rates are up to 77% and 76.2%, respectively [1]. However, the ASC with a simple structure occupies less space and can thus only cause minimal skin irritation and pain when the elbow joint undergoes early movement, decreasing the removal rates. (2) Nonunion is also a complication that needs to be avoided, and in some patients, the interfragmentary compression is significant for union. The traditional compression that internal fixation produces is discontinuous, static, and passive, and similarly to TBW, it only produces compression when the elbow is extended between 30 and 120° and against gravity [38], easily resulting in delayed union [39]. Instead, compression of the ASC derived from the compression arms in the device provides evenly distributed, continuous compression forces that can be transmitted across the fracture terminus, contributing to healing. (3) The ASC is manufactured using a Ni-Ti shape-memory alloy. Titanium and its alloys can become tightly integrated into the bone, which is a procedure that has been widely used in orthopedics [14]. Additionally, the ASC possesses a high strength and low modulus, which can effectively avoid the “stress shielding effect” [40]. (4) In clinical practice, surgeons prefer TBW for simple displaced olecranon fractures and plate fixation for displaced comminuted olecranon fractures [7]. However, when the fracture lines are irregular, the placement of fixators will be complex. Instead, the use of ASC is more flexible and widely applicable to diverse fracture lines. (5) As an important sports joint that is important for functional motion, the stability of the fixation depends mainly on whether early functional exercise can be achieved. We can adjust the number and size of the ASCs during the operation according to the needs of stability. Therefore, the patient can be encouraged to flex and extend the elbow joint earlier after the operation, which can explain the results of joint mobility of the patient in our study that was ideal. (6) The surgical procedure is also an important factor for choosing the treatment plan. The ASC technique is quite simple and only requires towel forceps and Kirschner wires for temporary fixation and an electric drill for drilling holes. When the ASC needs to be removed, it is treated with ice water to enhance its plasticity and then pulled out directly using tools. A simple procedure means a small amount of damage, a short operation time, and a low risk of vascular injury and blood loss.

However, several limitations should be highlighted: Due to the nature of the shape-memory alloy, the ASCs should be washed as needed with warm water for all subsequent procedures following implantation. Washing the implanted device with water at < 40 °C should be avoided because this may impede the fixation performance; in severe comminuted fractures, particularly in those with bone loss or subchondral fragments at the articular surface, initiating early movements after the ASCs osteosynthesis alone may cause failure. The ASCs may serve as an additional support to provide axial and angular stability based on the locking plate and screw fixation. Thus, the safety and stability of the application of the ASCs in bone-loss patients require further exploration. Additionally, before the study, we did not evaluate the patient’s bone health in detail and excluded factors such as osteoporosis that affect fracture healing. Due to the number of included patients, no patient was aged older than 60 years, a finding that may not accurately evaluate the application of the ASCs in patients with on old olecranon fractures. Additionally, although we achieved relatively successful outcomes after the follow-up of 57 patients, the study is a single-case-series retrospective study with no comparative cohort in our study. Thus, a further prospective cohort study is warranted.

## Conclusion

Accurate repair of the joint surface and absolute stability that allows the initiation of early joint motion are necessary for successful outcomes. Although our study was limited by the lack of a comparative group, the results of our series suggest reliable treatment outcomes. In the treatment of olecranon fractures, the ASC may not only serve as a favorable device for multi-fragmented fractures with rare hardware irritation but also provide a continuous compression to accelerate osseous healing, thereby aiding the restoration and permitting an early rehabilitation with a low incidence of postoperative complications.

## Supplementary information

**Additional file 1:** **Video 1.** this is the working principle of the ASC. The ASC was placed in 0-4 °C ice water for cooling in advance and then the ASC will be so malleable that the arms can easily be unfolded using needle forceps. When 40-50 °C water was used to warm the device by injector, its memory mechanical functions was stimulated, the ASC returned to his original form, which can provide a continuous lateral compressive force.

## Data Availability

We state that data will not be shared because all raw data were used to prepare the figures included in the article.

## Abbreviations

ASC: Arched shape-memory connector
TBW: Tension band wiring
PF: Plate fixation
